# Patient and Public Involvement am Deutschen Zentrum für Psychische Gesundheit: Erreichtes und Herausforderungen

**DOI:** 10.1007/s00115-024-01630-8

**Published:** 2024-03-20

**Authors:** Silke Lipinski, Ulrike Sünkel, Christina Totzeck, Thomas Dresler, Irina Baskow, Myriam Bea, Rüdiger Hannig, Isabel Dziobek

**Affiliations:** 1 Trialogischer Zentrumsrat des DZPG, German Center for Mental Health (DZPG), https://www.dzpg.org/partizipation/beteiligungsmoeglichkeiten; 2 Klinische Psychologie Sozialer Interaktion, Institut für Psychologie, Humboldt-Universität zu Berlin, Unter den Linden 6, 10099 Berlin, Deutschland; 3 Aspies e. V., Berlin, Deutschland; 4 Klinik für Psychiatrie und Psychotherapie, Universitätsklinikum Tübingen, Tübingen, Deutschland; 5 Forschungs- und Behandlungszentrum für psychische Gesundheit (FBZ), Ruhr-Universität Bochum, Bochum, Deutschland; 6 LEAD Graduate School & Research Network, Universität Tübingen, Tübingen, Deutschland; 7 Klinik für Psychiatrie und Psychotherapie, Charité Universitätsmedizin Berlin, Berlin, Deutschland; 8 ADHS Deutschland e. V., Berlin, Deutschland; 9 Bundesverband der Angehörigen psychisch erkrankter Menschen e. V., Bonn, Deutschland

**Keywords:** Partizipation, Trialog, Patientenbeteiligung, Angehörigenbeteiligung, Partizipative Forschung, Participation, Trilogue, Patient participation, Relatives participation, Participatory research

## Abstract

**Hintergrund:**

Patient and Public Involvement (PPI) beschreibt die Partizipation von Betroffenen und Angehörigen, d. h. Erfahrungsexpert:innen (EE), am Forschungsprozess. PPI ist in Deutschland in der Medizin und klinischen Psychologie wenig verbreitet, eine institutionelle Verankerung fehlt bislang. Die deutschlandweite und zentrumsübergreifende Umsetzung von PPI ist eines der Hauptziele des sich seit Mai 2023 im Aufbau befindenden Deutschen Zentrums für Psychische Gesundheit (DZPG). Partizipation von EE soll im DZPG auf allen Ebenen der Entscheidungsfindung implementiert werden.

**Ziele:**

Der Artikel beschreibt die Anfänge, Entwicklung und Herausforderungen der Umsetzung von Partizipationsstrukturen und -projekten im DZPG. Das zentrale politische PPI-Gremium im DZPG, der Trialogische Zentrumsrat (TZR), hat noch vor dem Beginn der finanziellen Förderung des DZPG in fast dreijähriger Arbeit eine umfassende PPI-Strategie für das DZPG entwickelt. Die Strategie sieht u. a. vor, eine weitreichende Mitsprache für EE in allen Entscheidungsgremien des DZPG zu implementieren, EE als Reviewer in die Begutachtung von Forschungsanträgen einzubeziehen, partizipative Elemente in alle Studien des DZPG zu integrieren und nutzerinitiierte Studien zu fördern. Die Implementierung der Strategie wird durch eine zentrumsübergreifende PPI-Infrastruktur, das Center for PPI, und die wissenschaftlichen PPI-Referent:innen gewährleistet. Zu den Aufgaben des Center for PPI gehört die Unterstützung der Mitsprache der EE sowie u. a. die Entwicklung von Instrumenten und Leitfäden für partizipative Forschung, die Zusammenführung von EE und Forschenden für gemeinsame Projekte sowie die Dokumentation und Qualitätssicherung für Partizipative Forschung.

Zu den besonderen Herausforderungen für die erfolgreiche Umsetzung der PPI-Strategie gehört die geringe Erfahrung mit PPI in Deutschland im Bereich der psychischen Gesundheitsforschung und weitestgehend fehlende strukturelle Implementierung. Derzeit erarbeitete Lösungsstrategien umfassen z. B. Schulungen für Forschende und EE, um die Vorteile und Wege zur Realisierung von PPI zu vermitteln und so gemeinsame Entscheidungsfindung und Forschung zu ermöglichen. Außerdem werden weitreichend der Zugang zu Wissen und Ressourcen für EE geschaffen und einheitliche Vergütungsregeln für EE erarbeitet.

**Schlussfolgerungen:**

Eine PPI-Strategie am DZPG wurde erfolgreich erarbeitet und wird derzeit durch die zentrumsübergreifende Infrastruktur Center for PPI implementiert.

## Abstract (en)

**Background:**

Patient and public involvement (PPI) describes the participation of patients and relatives, i.e., experts by experience (EE), in the research process. The PPI has not been widely adopted in the fields of medicine and clinical psychology in Germany and there is a notable absence of institutional support. The German Center for Mental Health (DZPG), which has been under construction since May 2023, aims to achieve nationwide and cross-center implementation of PPI, constituting one of its primary objectives. Participation of EE is to be implemented in the DZPG at all levels of decision-making.

**Objectives:**

The article describes the origins, development and challenges associated with the implementation of participation structures and projects in the DZPG. The central political PPI committee in the DZPG, the Trilogue Center Council (TZR), developed a comprehensive PPI strategy for the DZPG in almost 3 years of work, before the beginning of the financial support of the DZPG. Among various measures, the strategy entails establishing a far-reaching representation for EE in all decision-making bodies of the DZPG, to involve EE as reviewers in evaluating research proposals, to integrate participatory elements into all studies of the DZGG and to foster user-initiated research endeavors. The implementation of the strategy is ensured by a cross-center PPI infrastructure, the *Center for PPI*, and scientific PPI consultants. The Center for PPI’s tasks include supporting the voice of the EE and developing instruments and guidelines for participatory research, bringing together EE and researchers for joint DZPG projects as well as the documentation and quality assurance for participatory research. One of the particular challenges for the successful implementation of the PPI strategy is the limited experience with PPI in Germany in the field of mental health research and the widespread lack of structural implementation. Currently developed solution strategies include training for researchers and EE to communicate the benefits and pathways in the realization of PPI and thus enable shared decision-making and research. In addition, extensive access to knowledge and resources for EE will be created and uniform remuneration regulations for EE will be developed.

**Conclusion:**

A PPI strategy at the DZPG has been successfully developed and is currently being implemented by the cross-center infrastructure Center for PPI.

## Das DZPG setzt auf die Beteiligung von Betroffenen und Angehörigen

Psychische Erkrankungen beginnen meist früh, haben häufig langfristige Verläufe und gehen mit individuellem Leid einher. Daten zeigen, dass ihr Auftreten häufig ist und sie zu den Volkskrankheiten mit wachsender Krankheitslast zählen. Das Deutsche Zentrum für Psychische Gesundheit (DZPG) wurde mit dem Ziel gegründet, Forschungsbedingungen zu verbessern und versorgungsrelevante Ergebnisse schneller in die Praxis zu übertragen. Dabei werden insbesondere die gesamte Lebensspanne, Umweltfaktoren und die Entwicklung innovativer präventiver und therapeutischer Werkzeuge adressiert, um eine verbesserte Versorgung psychisch erkrankter Menschen zu erreichen. In einer historisch von Machtmissbrauch belasteten psychiatrischen und psychotherapeutischen Forschung und Versorgung sind Rechte und Mitsprache für betroffene Menschen von besonderer Bedeutung. Im DZPG werden bereits seit der Konzeptentwicklungsphase Perspektiven und Entscheidungen von Erfahrungsexpert:innen (EE) im Rahmen von Patient and Public Involvement (PPI) auf trialogische Weise integriert. PPI stützt sich auf eine Vielzahl partizipativer Forschungstraditionen aus verschiedenen Ländern und Zeiten (einen Überblick bietet Wright [22]). Rahmenwerke für PPI in der Gesundheitsforschung gibt es viele: In einer Übersichtsarbeit identifizierten Chudyk et al. [3] 14 Modelle mit 18 sich überschneidenden und 57 unterschiedlichen Elementen.

Patient and Public Involvement (PPI) findet in Deutschland bisher in Gesundheits- und Forschungsstrukturen punktuell statt und mit stark variierendem Maß an Mitspracherechten seitens der EE. In den letzten Jahren wurden auch in Deutschland vermehrt EE bei Leitlinienentwicklungen involviert; siehe z. B. Beteiligung in der S3-Leitlinie für psychosoziale Therapien, Autismus und bipolare Störungen. Außerdem gibt es Fachgesellschaften wie die Deutschen Gesellschaft für Bipolare Störungen (DGBS) oder die Wissenschaftliche Gesellschaft Autismus-Spektrum (WGAS), die z. T. seit vielen Jahren erfolgreich trialogisch arbeiten.

Patient and Public Involvement im DZPG baut auf eine Vielzahl internationaler und nationaler Vorläufermodelle und Ansätze (z. B. Trialog) auf. In Deutschland ist jedoch die strukturelle Verankerung von Mitentscheidungs- und gemeinsamer Forschungsaktivität auf allen Ebenen innerhalb einer großen Forschungsstruktur ohne Vorläufer.

Erstmalig wirken mithilfe einer umfassenden PPI-Infrastruktur EE auf allen Entscheidungsebenen eines Deutschen Zentrums für Gesundheitsforschung mit und partizipative Ansätze werden systematisch in allen Forschungsprojekten des DZPG gefördert.

„Das DZPG bedeutet für mich die Chance, das Ungleichgewicht und die Unterscheidung zwischen Arzt und Patient zu minimieren. Ich bin Teil des DZPG, weil ich hier für eine bedürfnisorientierte Therapie auf Augenhöhe kämpfen und mich für die partizipative Forschung einsetzen kann, um aktuellen Betroffenen eine qualitativ verbesserte Behandlung zu ermöglichen. Bei jedem Beitrag, den ich einbringe, sehe ich nicht nur alle Betroffenen und Angehörigen hinter mir, für die ich sprechen darf, ich sehe auch meine Kinder, die vielleicht auch mal eine Behandlung brauchen und die bestmögliche Versorgung verdienen.“ (Mitglied des Trialogischen Zentrumsrats)

### Partizipation in der Forschung

Menschen, die von einem bestimmten gesundheitsbezogenen Problem betroffen sind, sei es direkt oder als Angehörige, spielen eine zentrale Rolle in der Gesundheitsforschung. Häufig werden sie im Forschungsprozess jedoch lediglich als Proband:innen mitgedacht. Dabei können Menschen, die mit einer Erkrankung leben oder Betroffene unterstützen, ihre eigene Erfahrung auf vielfältige Weise und in allen Phasen des Forschungsprozesses gewinnbringend einbringen.

#### Infobox Begriffliches

Im Kontext von Beteiligung im Sinne des Patient and Public Involvement (PPI) werden die Begriffe „Patient“ und „Öffentlichkeit“ („Public“) teilweise als Sammelbegriff für direkt Betroffene und unter anderem auch Angehörige sowie Bürger:innen verwendet, z. B. in ihrer Rolle als später potenziell Betroffene oder im Zusammenhang von Forschung zu den Auswirkungen von Umwelteinflüssen auf die psychische Gesundheit. Es gibt eine Reihe von Begriffen und Diskussionen, wie die unterschiedlichen Stakeholder im Beteiligungsprozess bezeichnet werden können/wollen.^1^

Nach kritischer Reflexion haben sich die Akteur:innen im DZPG auf die Bezeichnung Erfahrungsexpert:in geeinigt. Sie schließt insbesondere Personen ein, die persönliche Erfahrungen mit psychischen Gesundheitsproblemen oder psychischen Erkrankungen oder Erfahrungen als Angehörige eines betroffenen Menschen im engen Umfeld gemacht haben. Unter dem Schirm dieses Begriffs werden die aus Eigenerfahrung und gegenseitiger Unterstützung gewonnenen Wissensbestände von Betroffenen wie Angehörigen als eigene Expertise erfasst.

„Partizipative Gesundheitsforschung“ versteht die Durchführung von Forschung als Koproduktion verschiedener Akteur:innen, um Veränderungen anzustoßen, die die gesundheitliche Chancengleichheit und das Wohlbefinden der Menschen fördern. Insbesondere die kontinuierliche Reflektion von Machtstrukturen zeichnet diesen Ansatz aus (PartNet [14]). PPI hat seinen Ursprung in Großbritannien und hat sich international vielerorts in der Gesundheitsforschung erfolgreich etabliert. PPI verfolgt das Ziel, idealerweise von der Antragstellung bis zur Veröffentlichung der Ergebnisse, nicht mehr nur ÜBER Erfahrungsexpert:innen, sondern MIT ihnen gemeinsam zu forschen (siehe INVOLVE [11]). Dabei stehen kollaborative Interaktion, bedeutungsvoller Austausch und Einbezug von Perspektiven über die des Forschenden hinaus im Zentrum des Prozesses.

**Figure 1:**
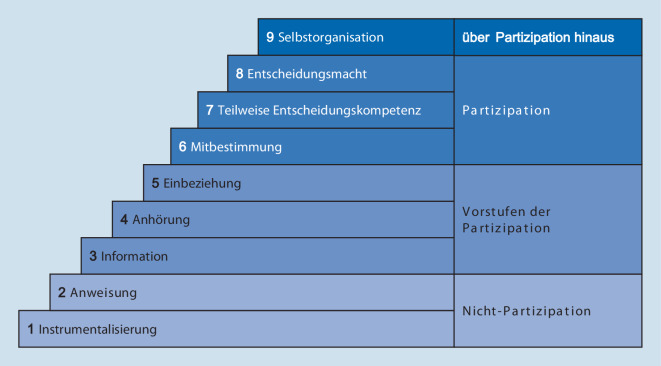

Ein bekanntes Stufenmodell, das die Ebenen der Beteiligung in der Gesundheitsforschung beschreibt, kommt aus der partizipativen Gesundheitsforschung (Abb. 1; [23]).

Die partnerschaftliche Einbindung von Betroffenen und Angehörigen in die Forschung zu psychischer Gesundheit ist ethisch und rechtlich geboten [27], kann die Versorgungsqualität verbessern und zu bedarfsgerechten Versorgungsangeboten beitragen (z. B. Dziobek und Lipinski [5]), da EE eine einzigartige Perspektive und Empathie für die Herausforderungen und Sorgen haben, mit denen Menschen mit psychischen Erkrankungen und Gesundheitsproblemen konfrontiert sind. Aus der Forschung zu PPI ist bekannt, dass Mitsprache von EE Forschung qualitativ besser, relevanter, z. T. sogar kostengünstiger machen und die Horizonte von Professionellen erweitern kann [9]. Daher fördern und fordern z. B. Regierungsrichtlinien im Vereinigten Königreich (UK; [7]) PPI in allen Phasen des Forschungsprozesses, um die Relevanz und Qualität der Forschung zu verbessern. Auch wissenschaftliche Fachzeitschriften machen zunehmend zur Bedingung, dass die eingereichten Arbeiten Beteiligung von EE vorweisen (*The BMJ* [20]).

### Entwicklung der Beteiligung von Erfahrungsexpert:innen am DZPG

In der psychologisch-psychiatrischen Forschung wird PPI mit echter Entscheidungsmacht der EE-Akteur:innen in Deutschland bisher kaum realisiert [5, 15]. Jedoch werden partizipative Ansätze bei Förderbekanntmachungen im Bereich Gesundheitsforschung immer häufiger vorgesehen und finanziell unterstützt. Auch in der Ausschreibung zum DZPG wurde dies entsprechend verortet („Richtlinie zur Förderung der Konzeptentwicklung zum Aufbau eines Deutschen Zentrums für Psychische Gesundheit“ vom 03.07.2020, [2]), indem das Bundesministerium für Bildung und Forschung (BMBF) die Antragstellenden beauftragte, die Beteiligung von EE „in allen Phasen der Planung, der Durchführung und der Umsetzung der Forschungsergebnisse“ zu verwirklichen. Zudem sollte eine „zentrumsübergreifende Strategie, die die geplanten partizipatorischen Prozesse und Elemente des Zentrums beschreibt“, ausgearbeitet werden. Ziel ist es, zu gewährleisten, dass die Bedürfnisse von EE angemessen berücksichtigt werden und durch die partnerschaftliche Beteiligung von EE an der Planung, Durchführung und Auswertung medizinischer Forschungsprojekte dazu beizutragen, Forschung qualitativ zu verbessern, die Relevanz ihrer Ergebnisse zu erhöhen und die Bearbeitung von Fragestellungen voranzutreiben, die für Betroffene und Angehörige von Relevanz sind. In der Größenordnung eines Deutschen Zentrums für Gesundheit gab es bezüglich des Aufbaus und der Gestaltung einer Zusammenarbeit von EE und Forschenden sowie der strukturellen Implementierung von PPI, wie sie für das DZPG bereits ab der Konzeptentwicklungsphase geplant war, in Deutschland bisher keine Vorgänger.

Zur konkreten Umsetzung wurde im Sommer 2021, bereits vor Beginn der finanzierten Konzeptentwicklungsphase, der Trialogische Zentrumsrat (TZR) gegründet, in welchem die Pluralität der Meinungen, Ideen, Erfahrungen und Perspektiven trialogisch (d. h. vonseiten 1. Betroffener, 2. Angehöriger und 3. Professioneller) miteinander verbunden werden. Der TZR setzte sich zunächst aus Mitgliedern bereits existierender (Klinik‑)Beiräte, Verbände und partizipativer Initiativen (z. B. Autismus-Forschungs-Kooperation [26]) zusammen. Die Mitglieder des TZR haben sich in ihrem eigens hierfür verfassten Mission Statement zum Ziel gesetzt, dass der Weg zu psychischer Gesundheit patientenorientiert und -sensibel gegangen, die Stigmatisierung psychischer Erkrankungen überwunden und Partizipation auf allen Ebenen vorangetrieben werden soll. Der TZR war vom ersten gemeinsamen Planungstreffen der Standorte an und in sämtlichen Arbeitsgruppen in die Konzeption der Infrastrukturen und Forschungsprojekte des DZPG eingebunden.

Parallel zur Mitarbeit an der Agenda des DZPG hat der TZR in der Konzeptentwicklungsphase die zukünftige PPI-Infrastruktur für das DZPG entworfen, d. h. es wurde konzipiert, wie die inhaltliche Zusammenarbeit (z. B. in Form von Gremienarbeit) gestützt, ausgebaut sowie Strukturen geschaffen werden können, die partizipative Forschung in größtmöglichem Umfang ermöglichen. Die so entstandene PPI-Strategie wurde mit im DZPG-Gesamtantrag verschriftlicht und letztendlich von Vertretenden des TZR in der Anhörung erfolgreich vorgestellt.

## Die PPI-Strategie am DZPG: politische Partizipation und partizipative Forschung

**Figure 2:**
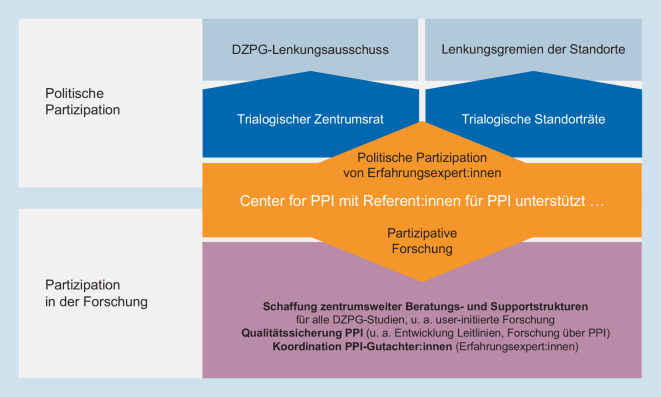

Um die partizipativen Strukturen und Abläufe innerhalb des DZPG zu gewährleisten, wurde eine zentrumsübergreifende PPI-Strategie entwickelt [4]. Diese fokussiert sowohl auf politische Partizipation zur Gewährleistung inhaltlicher Mitgestaltung als auch auf partizipative Forschung (Abb. 2).

### Politische Partizipation von Erfahrungsexpert:innen: die Trialogischen Räte

An den DZPG-Standorten sind bereits oder werden derzeit lokale Trialogische Standorträte eingerichtet, die sich regelmäßig treffen. Hier tauschen sich Betroffene, Angehörige und Forschende miteinander aus und organisieren gemeinsam Prozesse der inhaltlichen Mitgestaltung und Beteiligungsmöglichkeiten in Forschungsprojekten. Unter den EE der Trialogischen Standorträte sind nahezu alle großen Diagnosegruppen im Bereich psychischer Erkrankungen vertreten. Viele Mitwirkende haben langjährige Erfahrungen im Bereich der Selbsthilfe und der Selbstvertretung sowohl auf lokaler als auch auf Landes- und Bundesebene und haben bereits in (über)regionalen Gremien oder an Leitlinien der Arbeitsgemeinschaft der Wissenschaftlichen Medizinischen Fachgesellschaften (AWMF) mitgearbeitet. Jeder der sechs Trialogischen Standorträte wählt jeweils maximal zwei Vertreter:innen für Betroffene, Angehörige und Forschende als Sprechende; aus diesen setzt sich der Trialogische TZR zusammen, der somit bis zu 36 Personen umfasst.

Der TZR arbeitet kontinuierlich als eigenständiges Gremium sowie mit Stimmrecht in allen DZPG-Gremien an der wissenschaftlichen Strategie auf politischer Ebene mit, z. B. bei der Auswahl von Forschungsschwerpunkten und der Verankerung partizipativen Forschens in der klinischen Psychologie und Psychiatrie. Auch eine eigene „nutzergeleitete“ Studie wurde initiiert und befindet sich derzeit in der Umsetzung (siehe unten).

Ein Anliegen des DZPG ist es, die gesamte Lebensspanne zu berücksichtigen. Um dieses Ziel auch in der Partizipation zu erreichen, sprich auch die Perspektive und Erfahrung von Kindern und Jugendlichen einzubinden, wurden am Standort Bochum/Marburg sowohl der erste Kinderrat (6–12 Jahre) als auch der erste Jugendrat „Psychische Gesundheit“ (12–19 Jahre) gegründet. Beide sind bereits in die Prozesse des DZPG eingebunden und begleiten Forschungsprojekte, die vor allem Kinder und Jugendliche adressieren. Außerdem ist der TZR am Brückenschlag zum geplanten Deutschen Zentrum für Kinder- und Jugendgesundheit (DZKJ) beteiligt.

#### Beispiele für die Arbeit des Trialogischen Zentrumsrats

Nachdem der TZR im Juni 2021 zunächst als „Betroffenen- und Angehörigen-Beirat“ gestartet war, entwickelte sich intern schnell ein Bewusstsein dafür, dass echte Partizipation über einen nur beratenden, aber nicht entscheidenden „Bei“-Rat hinausgeht (Abb. 2) und dass im Sinne des trialogischen Gedankens auch Forschende Teil des Rats sein sollten. Daraus resultierte die Bezeichnung „Trialogischer Zentrumsrat“ – ein Rat mit Stimmrecht und fester Etablierung in der DZPG-Governance-Struktur. Aufgrund der teilweise unterschiedlichen Perspektiven von Betroffenen und Angehörigen haben beide Gruppen im Management Board jeweils eine Stimme. Dadurch hatte der TZR in der Projektentwicklungsphase im Management Board einen Stimmanteil von 25 %; durch die Hinzunahme der Gruppe der „Early Career Scientists“ in die Governance-Struktur seit der Projektförderphase liegt der Stimmanteil des TZR aktuell bei 22 %.

Über die Bezeichnung der Räte hinaus wurde vom TZR der Name des DZPG mitgestaltet. Zu Beginn der Konzeptphase war für das neue Gesundheitszentrum das Akronym „DZP“ vorgesehen. Dieses stellte sich als missverständlich heraus, sodass immer wieder von einem „Deutschen Zentrum für Psychiatrie“ oder einem „Deutschen Zentrum für Psychische Störungen“ die Rede war. Der TZR hat sich aus salutogenetischer Perspektive erfolgreich beim BMBF dafür eingesetzt, das „G“ für „Gesundheit“ in DZPG zu verankern. Aus Sicht des TZR ist ein wichtiges Ziel des DZPG, Forschung durchzuführen und zu fördern, die möglichst nachhaltig die psychische Gesundheit möglichst vieler Menschen in Deutschland verbessert.

Im Rahmen des DZPG werden den Lebensverlauf begleitende Studien durchgeführt und erhobene Studiendaten sollen auch mehrfach und für verschiedene Fragestellungen genutzt werden. Für die Einwilligung in die Datennutzung wird häufig ein „Broad Consent“ eingeholt, der nach einmaliger Zustimmung der Studienteilnehmenden eine breite und langfristige Datennutzung für Studienzwecke auch über die aktuelle Studie hinaus ermöglicht. Der TZR setzt sich dafür ein, dass der „Broad Consent“ zu einem „Dynamic Consent“ erweitert wird. Ein solcher ermöglicht eine Einwilligung für den aktuellen Nutzungszweck sowie eine separate Einwilligung der Datengeber:innen für jeden weiteren Nutzungszweck, für den sie jedes Mal kontaktiert werden müssen (z. B. über eine App). Hierbei sind sie in geeigneter Form über Ziel und Zweck der neuen Datennutzung aufzuklären und können dann entscheiden, ob ihre Daten dafür verwendet werden dürfen (z. B. [8, 19]). Der „Dynamic Consent“ kann durch die Notwendigkeit klarer Nutzenspezifikation und kontinuierliche Interaktion und Kommunikation zwischen Studienteilnehmenden und Datennutzenden Vertrauen erzeugen und die Bereitschaft erhöhen, sich für Forschung zu öffnen.

Als die Inhalte der Forschungslinien des DZPG definiert wurden, wirkten Mitglieder des TZR in sämtlichen Arbeitsgruppen mit und setzten sich dafür ein, dass Themen wie Peer-Support, Entstigmatisierung, Salutogenese, Umgang mit Betroffenen und Angehörigen sowie partizipatives Forschen in der klinischen Psychologie und Psychiatrie einen festen Platz in der Forschungsagenda der kommenden Jahre in Deutschland haben. Zudem hat der TZR eine selbstgeleitete Studie angestoßen, in der Forschungsprioritäten aus Sicht von EE erhoben werden (siehe unten).

### Partizipation von Erfahrungsexpert:innen auf Ebene der Forschung

Um sowohl ethischen und rechtlichen Anforderungen zu entsprechen als auch um die vielfältigen positiven Effekte partizipativer Forschung im DZPG zur Entfaltung zu bringen, sollen EE im DZPG umfänglich in die konkrete Forschung integriert werden, d. h. es soll sichergestellt werden, dass EE aktiv an der Konzeption, Umsetzung, Evaluation und Dissemination von Forschungsprojekten beteiligt werden und Entscheidungskompetenzen haben und wahrnehmen können. Die konkrete Themenwahl, Mitbestimmung bei Outcomevariablen, gemeinsame Erstellung von Fragebögen, Verringerung der Belastung von Studienteilnehmenden bei Experimenten, die gemeinsame Präsentation von Ergebnissen auf Fachtagungen und die Entwicklung geeigneten Informationsmaterials für verschiedene Zielgruppen sind Beispiele hierfür.

Da auch Kinder und Jugendliche Zielgruppe der Forschung des DZPG sind, gehört es dazu, auch sie an der Planung und Umsetzung dieser Studien aktiv zu beteiligen. Lange Zeit wurden die Aussagen von Minderjährigen nicht ernst genommen und ausschließlich Eltern einbezogen. Dabei sind die Auskünfte von Kindern und Jugendlichen genauso wichtig und reliabel [12, 17]. Ihre Partizipation in der psychischen Gesundheitsforschung in Deutschland entwickelt sich noch langsamer als bei den Erwachsenen. Daher werden im DZPG gemeinsam mit Kindern ab dem Grundschulalter über die Jugend bis hin zum jungen Erwachsenenalter neue Herangehensweisen entwickelt, um auch in der partizipativen Forschung die gesamte Lebensspanne einzubeziehen.

### Nutzerinitiierte Studie

Über die partizipative Forschung hinaus werden im DZPG auch eigenständige, sog. nutzerinitiierte sowie nutzergeleitete Studien realisiert. Der TZR hat als Thema der ersten selbstinitiierten Studie eine Erhebung von Forschungsprioritäten aus Sicht von EE gewählt. Um die von EE gesehenen Forschungsnotwendigkeiten in Deutschland zukünftig auf einer noch breiteren Basis berücksichtigen zu können, werden in einem deutschlandweiten mehrschrittigen partizipativen Prozess Menschen mit Erfahrung im Bereich psychischer Erkrankungen zu ihren Bedürfnissen und Wünschen bezüglich Forschungsthemen befragt. Für das Beteiligungsvorhaben wird gemeinsam eine an die Zielgruppe angepasste Plattform konzipiert, auf der verschiedene Beteiligungsmodule als „Onlinedialog“ aufgesetzt werden. Die ermittelten Bedarfe an aufzugreifenden oder zu verstärkenden Forschungsthemen werden zu einem „Kompass“ der Forschungsprioritäten von EE, durch den sichergestellt werden wird, dass das DZPG auf die Bedürfnisse und Perspektiven, der von der Forschung direkt betroffenen Personen eingeht.

### „Center for PPI“: standortübergreifende Infrastruktur für Teilhabe und Partizipation von Erfahrungsexpert:innen

Um die sowohl politische Partizipation als auch partizipative Forschung umfassende PPI-Strategie, wie oben in Auszügen beschrieben, im DZPG flächendeckend auf hohem Niveau zu ermöglichen und Forschende und EE allerorts gleichermaßen bestmöglich bei der Zusammenarbeit zu unterstützen, wurde eine zentrumsübergreifende Infrastruktur für unumgänglich befunden und seit Beginn der Förderphase aufgebaut.

Analog zu anderen Forschungsinfrastrukturen, also standortübergreifenden Strukturen z. B. für die Bereiche Klinische Studien, Neuroimaging oder Psychotherapie, wurde die Infrastruktur für PPI, das „Center for PPI“, dauerhaft im DZPG verankert. Damit verbunden war auch die Schaffung von Personalstellen für PPI-Referent:innen an den Standorten und in der Geschäftsstelle, die idealerweise mit Personen zu besetzen sind, die eine „Doppelqualifikation“ mitbringen, also sowohl als Betroffene oder Angehörige über Erfahrungsexpertise verfügen als auch einen wissenschaftlichen Hintergrund und Erfahrung im Bereich partizipativer Forschung haben.

„Als PPI-Referentin bin ich gespannt, wie Partizipation entwickelt und dann auch gelebt werden kann. Für mich ist das ein neues spannendes Feld, welches ich als essenziell und wichtig erachte. In diesem Feld liegt viel Potenzial, welches vollumfänglich ausgeschöpft werden sollte!“ (Mitglied des Trialogischen Zentrumsrats)

Das Center for PPI hat zwei Hauptaufgabenfelder:

Zum ersten Aufgabenfeld gehört die Entwicklung und Unterstützung der Arbeit der Standorträte und des Zentrumsrats, um Partizipation zu ermöglichen. Hierzu gehören u. a. Organisation von Online- und Präsenztreffen der Räte, Unterstützung bei der Entwicklung von Geschäftsordnungen auf lokaler und zentraler Ebene, in denen EE die Arbeit der Räte für sich selbst transparent und nachvollziehbar gestalten, Förderung der Kommunikation und Zusammenarbeit von EE und Forschenden im Kontext der gemeinsamen Gremienarbeit, Erarbeitung von Vergütungsregelungen und Aufbau eines Glossars.

Das zweite Aufgabenfeld besteht in der Unterstützung und Förderung partizipativer Forschung sowohl in zentrumsübergreifenden als auch standortspezifischen Projekten. Zu den Aufgaben gehören hier u. a. die Entwicklung und Vermittlung von Informationsmaterial und Leitfäden für Partizipation in DZPG-Projekten sowie Instrumenten und Methoden partizipativer Forschung (z. B. Austauschformate wie Fokusgruppen oder World-Café, (digitale) Tools für barrierefreie Zusammenarbeit, Plattformen für die Verbreitung von Ergebnissen an EE und die Allgemeinbevölkerung). Außerdem sind hier der Aufbau eines Netzwerks mit externen Expert:innen, Aufbau und Schulung von EE-Gutachter:innen für intramurale Förderprogramme, Beratung und Schulung von Forschenden u. a. anhand etablierter Instrumente wie der GRIPP2-Guidlines [18] zu nennen. Des Weiteren sollen Schulungsprogramme für Forschende und EE im engen Zusammenwirken mit der „DZPG Academy“ entwickelt werden.

Um gegenseitiges Lernen und Austausch zu ermöglichen, hat auch bereits die interdisziplinäre Zusammenarbeit des Centers for PPI mit den Patientenbeteiligungsstrukturen der anderen Deutschen Zentren für Gesundheitsforschung^2^ begonnen.

## Herausforderungen in der Umsetzung der PPI-Strategie und Lösungswege

Wenn PPI durch externe Anforderungen (z. B. Ausschreibungen für Forschungsgelder) motiviert werden soll, besteht immer auch die Gefahr des sog. „Tokenismus“, in dem EE lediglich oberflächlich und symbolisch beteiligt werden, ohne jedoch echte Beteiligungsmöglichkeiten und gleichberechtigtes Entscheidungsrecht zu haben [13]. Es besteht jedoch die ethische Verpflichtung durch PPI, für gerechte und transparente Machtverhältnisse zu sorgen [5]. Das ist das gemeinsame Ziel aller Beteiligten im DZPG.

In der nun mehr als drei Jahre währenden PPI-Aktivität für das DZPG, die zu einem Großteil noch vor dem offiziellen Förderbeginn erfolgte, konnte eine Mehrheit der unvermeidlichen Herausforderungen bereits erfolgreich genommen und für die Partizipation von EE viel erreicht werden. Für die besonderen Herausforderungen, die sich aus dem Ziel einer strukturellen Implementierung sowie der Aufgabe, PPI parallel für sechs unterschiedliche Standorte auszurollen, ergeben, werden auch zukünftig weiter tatkräftig Lösungsansätze durch das Center for PPI erarbeitet. Einige dieser Herausforderungen und Lösungsversuche sollen hier im Sinne eines „shared learning“ geteilt werden.

### Zeitliche Ressourcen von Erfahrungsexpert:innen

Seit Beginn der Arbeit am DZPG sind EE überall zur Mitarbeit eingeladen, jedoch sind die erheblichen dafür erforderlichen Zeitressourcen nur begrenzt vorhanden. Während die Arbeit am DZPG für die beruflich Forschenden zum Arbeitsauftrag gehört – das Pensum jedoch erheblich ist und oft außerhalb der üblichen Arbeitszeit realisiert wird – sind EE in der Regel ehrenamtlich und in ihrer Freizeit tätig. Insbesondere die zusätzlichen Aufgaben, die mit einer Sprecherschaft des TZR einhergehen, sind mit einem hohen Zeit- und Reiseaufwand verbunden, die viele EE kaum mit einer beruflichen Tätigkeit und/oder familiären Aufgaben vereinbaren können. Auch mitten am Tag liegende und kurzfristig angesetzte Termine erschweren für viele EE eine kontinuierliche Mitarbeit. Darüber hinaus benötigen EE häufig zusätzliche Zeit für Erörterungen, um Besprechungsinhalte zu verstehen und Tragweite und Konsequenzen anstehender Entscheidungen zu überblicken.

Um eine kontinuierliche und langfristige Mitarbeit von EE trotz Berufstätigkeit und Belastungen durch eigene psychische Erkrankungen oder die von Familienangehörigen zu ermöglichen, müssen die Bedingungen und Aufgaben dies berücksichtigen, und – wo es möglich ist – strukturelle und inhaltliche Unterstützung gewährleistet werden. Dazu gehört auch ein Erwartungsmanagement, das die Aufgaben und den zeitlichen Umfang klar kommuniziert. Diese Herausforderung wurde kürzlich im TZR dadurch adressiert, dass Satzungen entwickelt und konsentiert wurden, in denen die Arbeit der Räte festgelegt wurde, aber auch durch die Schaffung von Unterstützungsstrukturen wie die PPI-Referent:innen.

### Zugang zu Wissen und strukturellen Ressourcen

Erfahrungsexpert:innen haben in der Regel keine direkte Anbindung an eine Universität oder Forschungseinrichtung. Daher ist der Zugang zu Fachliteratur nur eingeschränkt möglich. Auch wenn ein Zugang ermöglicht wird, stehen jedoch viele EE, die z. B. eine Literaturrecherche durchführen wollen, ohne wissenschaftlichen Hintergrund vor der Barriere, komplexe Datenbankabfragesoftware bedienen und Recherche sowie Sichtung der Literatur zumeist in englischer Fachsprache durchzuführen zu müssen. Solche Probleme können aber in Teilen durch einfacheren Zugang zu Werkzeugen wie Übersetzungssoftware abgemildert werden. Erstrebenswert ist eine offizielle Anbindung von EE an eine Forschungseinrichtung mit Zugangsdaten zu Software und Onlinebibliotheken (z. B. durch einen assoziierten Status), sodass eine barrierefreie Nutzung auch von zu Hause aus jederzeit möglich ist. Die Bedarfe von EE werden am DZPG zunehmend erkannt und es wird an Lösungen gearbeitet, unterstützt durch das Center for PPI und die Referent:innen.

In gemeinsamen Treffen von Forschenden und EE ist es auch von Bedeutung, eine klare und verständliche Kommunikation zu gewährleisten, die unterschiedliche Wissensstände berücksichtigt, um sicherzustellen, dass alle Beteiligten angemessen mitarbeiten können. In diesem Sinne haben die Arbeiten an einem Glossar für wissenschaftliche Fachtermini und DZPG-spezifische Begriffe begonnen.

### Anerkennung Erfahrungsexpertise als Qualifikation bei Einstellungsprozessen

Obwohl in den letzten Jahren zunehmend Menschen mit eigenen Erfahrungen mit psychischen Erkrankungen als sog. Peers in psychiatrischen Kliniken und Einrichtungen eingesetzt werden und sich das neue Berufsbild des „Genesungsbegleiters“ etabliert [10], wird Psychiatrieerfahrung oder Erfahrung mit psychischer Krankheit bis heute überwiegend als Disqualifikation für traditionellere Berufe in psychiatrischen Kliniken oder klinisch-psychologischer Forschung (z. B. Ärzt:in, Pflegefachkraft) angesehen [16, 24, 25]. Diana Rose schreibt treffend [16]:

„Es ist schwierig, ein Gleichgewicht zu finden – dass eine Diagnose und Erfahrung mit Gesundheitsdiensten eine Qualifikation für die Stelle ist und nicht ein Handicap.“

Am DZPG soll eigene Erfahrung mit psychischer Erkrankung jedoch künftig als positives Auswahlkriterium („Erfahrungsexpertise“ im Sinne einer Doppelqualifikation) gewertet und bei Neueinstellungen wissenschaftlichen Personals positiv berücksichtigt werden. In einer kürzlich erfolgten Stellenausschreibung wurde Erfahrungsexpertise explizit als gewünschte Qualifikation genannt, und aktuell ist die Hälfte der PPI-Referent:innenstellen im DZPG mit doppelqualifizierten Personen besetzt.

### Finanzielle Rahmenbedingungen: Aufwandsentschädigungen und Ressourcen des Center for PPI

Ein komplexes Thema ist die Regelung angemessener finanzieller Aufwandsentschädigung von EE. Bislang gibt es bundesweit in etwa so viele Regelungen wie Einzelprojekte. Im DZPG müssen Lösungen für Anwendungsfälle mit ganz unterschiedlichen Dimensionen gefunden werden: Einerseits für kleinere Stundenumfänge z. B. im Rahmen eines einzelnen Projekts, aber auch vertragliche Konstrukte für EE, die als solche über längere Zeiträume und in größerem zeitlichem Umfang in Forschungsprojekten mitarbeiten. Daran schließt sich die Frage der Höhe der Aufwandsentschädigungen, respektive die (tarifliche) Eingruppierung von EE an, was auch die Frage der Berücksichtigung bereits erworbener beruflicher Qualifikationen (z. B. Hochschulabschluss vs. ohne abgeschlossene Berufsausbildung) einschließt.

In dieser Frage können selten die Erwartungen aller Beteiligten erfüllt werden. Für die kontinuierliche politische Partizipation wurde vom TZR – zumindest testweise – das Modell einer Pauschale gewählt. Natürlich kann eine einheitliche Aufwandsentschädigung mit unterschiedlich viel Engagement gefüllt werden und benachteiligt damit die besonders Engagierten. Darüber hinaus existieren teilweise Vorgaben und Verwaltungsabläufe, die eine Auszahlung regelmäßiger oder pauschaler Aufwandsentschädigungen sowie eine barrierefreie Auszahlung verhindern. Zusätzlich erschwerend ist, dass sich viele EE in Lebenssituationen befinden, in denen die Annahme von Honoraren oder Aufwandsentschädigungen für sie sogar zum Nachteil werden kann (z. B. Verlust von Rentenzahlungen, volle Anrechnung auf Sozialleistungen, Notwendigkeit einer Steuererklärung und Steuerberatung). Das Center for PPI erarbeitet derzeit in Zusammenarbeit mit den EE übergreifende Empfehlungen für das DZPG, auch um entsprechende Posten in Zukunft verlässlicher budgetieren zu können. Zudem soll dabei aufgezeigt werden, welche Fragen auch politischer Klärung bedürfen, um bez. der Aufwandsentschädigungen in der deutschen Partizipationslandschaft weiterzukommen.

Obwohl das Thema PPI im Vergleich zu anderen Themen des DZPG in der Öffentlichkeit überproportional viel Aufmerksamkeit erhält, ist die Finanzierung nur teilweise gesichert. In der Konzeptentwicklungsphase wurde PPI durch das BMBF umfänglich finanziert. Für den Zeitabschnitt zwischen Abgabe des Antrags und Beginn der Förderphase wurden jedoch keine externen Mittel bereitgestellt, um die gerade aufgebauten Strukturen, auf die man ab Förderbeginn wieder zurückgreifen sollte, zu erhalten. Um die Zusammenarbeit auch in der fast einjährigen Zwischenphase, in der die Vorarbeiten zum DZPG weiter vorangingen, kontinuierlich zu ermöglichen, sind die Standorte für Aufwandsentschädigungen, Reisekosten und administrative Unterstützung eingesprungen.

Auch wenn die Grundfinanzierung für PPI im DZPG nicht infrage steht, so befindet sich das Budget für PPI dennoch in Teilen im Wettbewerb zur Forschungsfinanzierung. Dabei werden stabile und langfristige finanzielle Rahmenbedingungen benötigt, um PPI am DZPG so ermöglichen zu können, wie in der erarbeiteten Strategie geplant. Auch konnten die Referent:innenstellen für das Center for PPI gleich zu Beginn durch DZPG-weite finanzielle Kürzungen des Mittelgebers nicht vollumfänglich realisiert werden. In europäischen Förderprogrammen steht das Budget für strukturelle PPI-Maßnahmen zumeist nicht in Konkurrenz mit dem Budget für die ausgeschriebene Forschung. Der TZR und das Center for PPI engagieren sich, das Thema immer wieder aufzugreifen und für eine angemessene und bedarfsorientierte Ausstattung zu werben.

„Ich wünsche mir, dass die gegenwertige Wertschätzung für PPI sich auch in den zugeteilten Geldern reflektiert, die unterstützende Infrastrukturen und partizipative Forschung dringend brauchen.“ (Mitglied des Trialogischen Zentrumsrats)

### Entscheidungen und Macht teilen

Traditionelle, oft paternalistisch-hierarchisch geprägte Strukturen haben im deutschen Gesundheitswesen und in der Wissenschaft Barrieren für eine Zusammenarbeit auf Augenhöhe errichtet, die in anderen Ländern bereits erfolgreicher abgebaut werden. Forschende, die noch nie partizipativ gearbeitet haben, haben häufig Schwierigkeiten mit der Vorstellung, künftig gemeinsam oder sogar partnerschaftlich Forschung mit EE zu planen und umzusetzen, wozu auch die Sorge gehört, Macht über den Forschungsprozess zu verlieren [5]. Jedoch haben auch Betroffenen- und Angehörigenvertreter:innen vereinzelt Schwierigkeiten, Augenhöhe einzufordern, weil sie selbst noch der Meinung sind, Forschung sei allein Sache der Professionellen, in die sie sich nicht einzumischen hätten. Diese Haltung kann als Folge historischer Vernachlässigung von Erfahrungswissen Betroffener und Angehöriger gesehen werden, die es durch PPI zu überwinden gilt. Des Weiteren erfordert PPI Zeit, u. a. durch gegenseitiges Lernen, Abstimmungsprozesse und benötigte Transparenz [5]. Eine zum Teil geäußerte Sorge von Forschenden ist, durch PPI im Forschungsprozess zeitlich aufgehalten zu werden.

Für eine gewinnbringende Zusammenarbeit und den Abbau von Berührungsängsten können Schulungen förderlich sein. Auch reichen persönliche Erfahrungen mit psychischer Erkrankung allein nicht immer für eine qualifizierte Mitarbeit aus. So ist z. B. für Gutachtertätigkeiten weitere Einarbeitung notwendig. Für Schulungen gibt es bereits Vorbilder auf nationaler und internationaler Ebene, z. B. die European Patients’ Academy on Therapeutic Innovation (EUPATI), die Patienten-Experten-Akademie (PEAK), siehe auch [6], und auch im Bereich psychischer Gesundheitsforschung [1]. Ein Ziel der PPI-Infrastruktur ist deshalb die Entwicklung eines „Curriculums für partizipative Forschung“ in Zusammenarbeit mit der „DZPG Academy“. Neben „Science Literacy“ können im Rahmen von Schulungen auch Angebote gemacht werden, die Empowerment von EE fördern, z. B. durch Trainings für Gremienmitarbeit, wie sie bereits erfolgreich im ERA-Net Neuron stattfinden, die EE die nötige Sicherheit geben können, sich in ungewohnten Kontexten zu trauen, Redebeiträge zu leisten. Ein weiterer wichtiger Ansatz ist die Intensivierung von Kommunikation zwischen Forschenden und EE, die durch regelmäßige Begegnungen in gemeinsamen Projekten und Gremien erzielt und durch gegenseitiges Vertrauen gefördert werden kann.

Dass die langfristige Investition in PPI ein Weg ist, der für alle Beteiligten einen eindeutigen Mehrwert bereithält, erleben wir bereits jetzt auf allen Ebenen im DZPG.

## Fazit für die Praxis

- Das Deutsche Zentrum für Psychische Gesundheit (DZPG) hat als eines seiner Hauptziele formuliert, einen nationalen Standard für die Partizipation von Betroffenen und Angehörigen über die gesamte Lebensspanne zu etablieren.
- Die Patient-and-Public-Involvement(PPI)-Strategie umschließt die Förderung von Partizipation sowie partizipativer Forschung. Beides wird durch die eigens geschaffene PPI-Infrastruktur („Center for PPI“) unterstützt.
- Trotz Herausforderungen wie der Sicherung echter Entscheidungsmacht und angemessener finanzieller Vergütung ist schon sehr viel erreicht: Die Beteiligten diskutieren überwiegend auf Augenhöhe miteinander und Partizipation gehört aktuell zu den Topthemen bei öffentlichen Veranstaltungen.

## Footnotes

1 Siehe zur Frage der Begriffe auch eine Publikation von PartNet [21] unter https://opus4.kobv.de/opus4-ash/frontdoor/index/index/searchtype/latest/docId/475/start/0/rows/10.

2 Für mehr Informationen siehe https://deutschezentren.de/patientenbeteiligung-datenreiche-medizin/.
